# The hidden complexity of the simple world of basic experimental psychology: the principal and practical limits of gaining psychological knowledge using the experimental method

**DOI:** 10.3389/fpsyg.2024.1397553

**Published:** 2025-04-11

**Authors:** Christof Kuhbandner, Roland Mayrhofer

**Affiliations:** Department of Psychology, University of Regensburg, Regensburg, Germany

**Keywords:** experimental psychology, experimental method, methodology, replication crisis, epistemology, complexity

## Abstract

Basic experimental research in psychology is based on the assumption that law-like behavior can be observed if the complexity of the human psyche is reduced by the creation of experimental settings in which simple psychical phenomena occur which reflect the effect of an isolated psychological mechanism. However, we show that this assumption does not hold for many phenomena studied in basic experimental psychology because even phenomena that are regarded as simple and fully controllable often fluctuate unpredictably as a function of unintentionally chosen details of the experimental setting. The reason is that in a complex system like the human psyche, even minimal, and from the perspective of the investigated research question irrelevant, differences in the experimental setting can build up to large unsystematic effects. Law-like behavior in experiments could only occur if truly low-level mechanisms were studied in a truly isolated way. However, this is often not the case in current experimental research. One problem is that often fuzzy theoretical terms are used which only give the impression that low-level mechanisms are being investigated, although in reality the complexity of the human psyche is unintentionally brought on board. Another problem is that, unlike in the natural sciences, the mechanisms of the human psyche can only be isolated from each other to a limited extent because the human psyche always reacts as a whole system. If such problems could be overcome, meaningful knowledge could be gained through experimental psychological research. However, the knowledge gained is very limited in terms of its explanatory power for human behavior, as it is only helpful for understanding a very specific aspect of behavior, namely the mechanistic functioning of isolated low-level mechanisms. When it comes to understanding motivated behavior in real life, knowledge about the non-mechanistic functioning of the higher levels of the human psyche is necessary, but this knowledge cannot be gained through the experimental method.

## 1 Introduction

One of the defining elements of any science is the method used to attempt to gain knowledge about the subject of research. A currently widespread methodological approach to gaining knowledge in the field of psychological science is the experiment, a method that has proven to be very fruitful in the field of natural sciences.^1^ Particularly in the field of basic experimental psychology, there is a prevailing conviction that general laws of the human psyche can be established by means of the experimental method, similar to the natural sciences. The aim of this article is to critically examine this conviction.

## 2 The goal of science

It is a commonly shared view that the goal of science is to develop knowledge that allows us to predict which phenomena will occur if certain conditions are present. The most fundamental prerequisite for the development of such knowledge is that regularities are observed when a phenomenon is explored. In the most basic sense, “regularity” means that a certain observation that is made when a certain condition is present is observed again when the same condition is present again. Only if this is the case, knowledge about the phenomenon can be gained in the sense that theories can be developed which allow to predict what will happen if certain conditions are present.

However, to understand the great success of science, it is important to realize that science strives to establish theories about the existence of a certain form of regularity. For example, the Encyclopedia Britannica defines “science” basically as follows:^2^

“In general, a science involves a pursuit of knowledge covering general truths or the operations of fundamental laws.”

According to such definitions, the ultimate goal of science is not to establish theories that predict the occurrence of phenomena at the level of a singular object, but to establish *general* theories that predict the occurrence of phenomena for many different objects. For instance, the goal of physics as a science is not to establish a theory that predicts what movement is observed when a specific apple falls from a specific tree, but to establish a *general* theory that describes the falling movement of any object anywhere on Earth. This goal is achieved by postulating a certain cause-and-effect mechanism at the level of a property that is shared by many different objects. The objects to which the theory applies can nevertheless all be unique because they can differ in other object properties about which the theory makes no statements.^3^

## 3 The experimental method as the basis for the successful establishment of general theories in the natural sciences

With regard to the goal of establishing general theories, impressive successes have been achieved in the field of natural sciences. For instance, in physics, several universal laws were established that appear to exactly predict for any object anywhere in the universe what will be observed if a certain condition is present, such as the law of thermodynamics or the four laws of force (Ulanowicz, 2018).

This success was by and large made possible by using a very specific method to empirically test the validity of a proposed general theory: the experimental method. The use of this method was necessitated by an epistemic problem that arises when attempting to empirically test a general theory. To examine whether the predictions of a general theory correctly describe the occurrence of phenomena, it is necessary to explore whether all objects that have the property for which the theory formulates a cause-effect mechanism behave as predicted by the theory. However, objects not only have the specific property for which the theory under investigation makes a prediction, but also other properties on which other cause-effect mechanisms operate than that specified in the theory under investigation.

Accordingly, if one simply observed the behavior of objects in real life, one could not validate whether the predictions derived from a certain cause-effect mechanism correspond to the observations made, because the observed behavior is always determined by the interplay of all cause-effect mechanisms that simultaneously operate on the various object properties. Due to this fact, general theories that predict a certain cause-effect relationship cannot be empirically validated in real-life situations. An illustrative example is the law of gravitation. According to the law of gravitation, gravity accelerates every object at exactly the same rate so that heavy and light objects should fall at exactly the same speed. However, if one simply drops a feather and a steel ball in real life, one will observe that this is not the case, which seems to refute the law of gravitation. The reason why feathers and steel balls fall at different speeds in real life is that, in addition to gravity, there is a second influencing factor: air resistance.

This epistemic problem made it necessary to develop a method that allows the cause-effect mechanism specified by a specific general theory to be examined in isolation from all other simultaneously operating cause-effect mechanisms. And this is exactly what is achieved by the experimental method, which consists of deliberately manipulating the cause specified in the theory under investigation and measuring the resulting effect, while at the same time trying to eliminate the effects of all other additionally operating cause-effect mechanisms. An illustrative example is the way in which it could be empirically demonstrated that the law of gravitation makes correct predictions. This was made possible by the fact that an experimental setting was created in which objects were only influenced by gravity and no longer by air resistance, which was achieved by letting different objects fall in a vacuum. And indeed, under such conditions, feathers and steel balls fall at exactly the same speed, as predicted by the law of gravitation.

## 4 The adoption of this scientific logic in the field of psychological science

**Figure 1:**
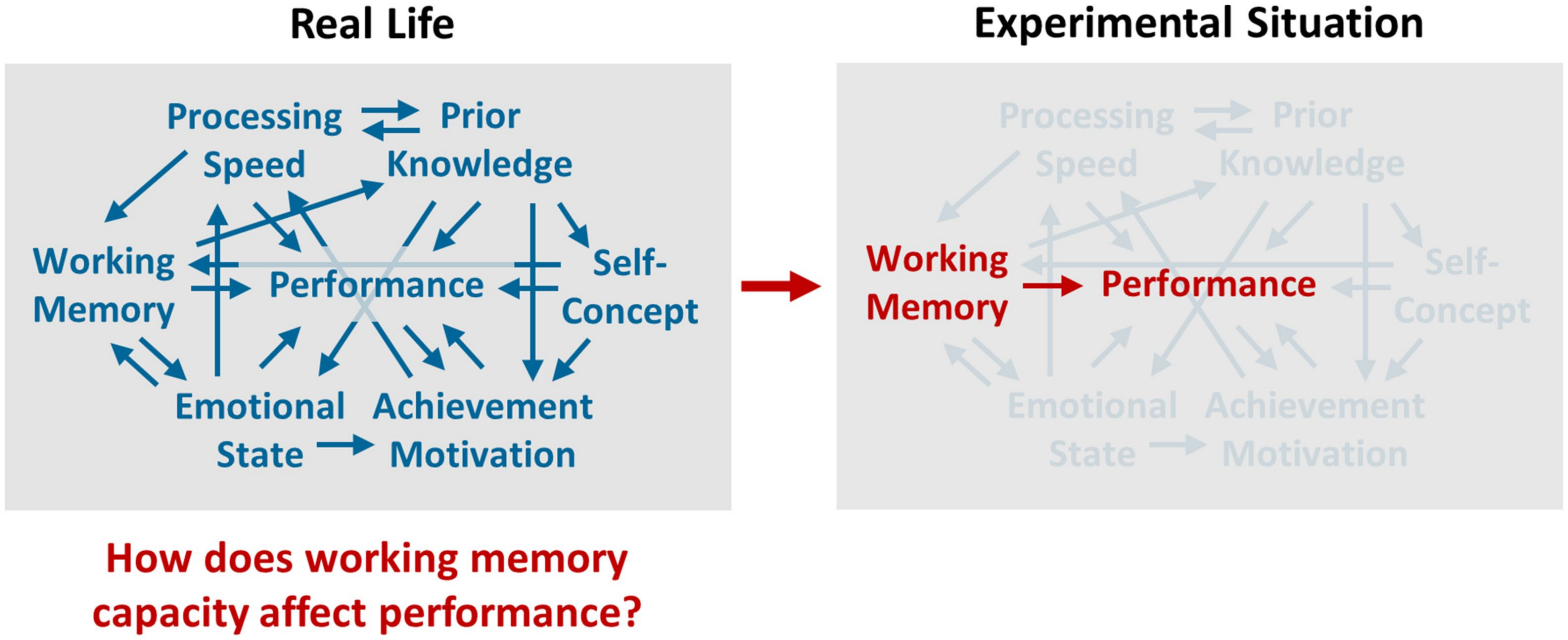
Illustration of the use of the experimental method in psychological research using the question of the relationship between working memory capacity and performance. In real life situations, performance is influenced by a variety of interacting cause-effect mechanisms at the same time such as those shown as examples on the left. In order to empirically examine whether there is a law-like relationship between working memory capacity and performance which can be described by means of a general theory, an experimental setting is created in which only the effect of working memory capacity influences performance, whereas the effects of all other cause-effect mechanisms, which are referred to as so-called confounding variables, are tried to be removed.

In view of the successes in establishing general theories by means of the experimental method in the field of natural sciences, the hope was raised that the same scientific logic can be applied in the field of psychological research where the aim is to predict the occurrence of psychical phenomena,^4^ and that comparable successes can be achieved (for an illustration of the use of the experimental method in psychological research, see Figure 1; for a description of the research history of the experimental method in the field of psychology, see Mandler, 2007).

Such a conviction is particularly common in the field of basic experimental psychological research, where attempts are made to gain knowledge about basic processes of the human psyche such as, for instance, perception or the storage of information. Characteristic of this field of research is the strong belief that law-like behavior is observed if the complexity of the human psyche is reduced by the creation of experimental settings in which simple psychical phenomena occur which reflect the effect of an isolated psychical mechanism. For instance, in an editorial of the journal *Experimental Psychology*, the editors describe the principles that characterize high-quality research as follows (Eder and Frings, 2018, p. 258):

“First, it should be noted that experimentation is the ‘golden standard’ of scientific knowledge seeking. Experiments provide insight into cause and effect by systematic investigation of what outcome occurs when a particular factor or variable is manipulated. (…) A strong experiment gives great confidence in the inference of a causal relationship among variables.”

And indeed, it is often claimed that it can be shown by means of basic experimental psychological research that certain psychical phenomena are governed by general laws. For instance, many articles and textbooks explain the course of forgetting of information stored in memory with recourse to a general law because there seems to be one retention function that describes the course of forgetting for many different types of memory as well as different memory contents (i.e., the power law of forgetting: the rate of decay slows with the passage of time; e.g., Rubin and Wenzel, 1996; Wixted and Ebbesen, 1991). Another example is the amount of information that can be held in working memory. It was proposed early on that there is a fixed number of items that humans can hold in working memory, with the suggestion that this number is 7 ± 2, which is frequently referred to as Miller’s Law (Miller, 1956).

## 5 A first limit for the establishment of general theories: probabilistic instead of invariable cause-effect relationships

However, it became apparent that there are obviously limitations to describing psychical phenomena using general theories. In classical physics, it is the case that a cause always produces the same effect for all objects for which a theory is valid when all other cause-effect mechanisms operating on an object are excluded. That is, causes are invariably followed by their effects. However, as it turned out, such invariable patterns of causations are typically not observed in experimental psychological studies. There, the psychical phenomena that are expected to occur if a certain cause is present according to a postulated psychological theory do not occur invariably when the cause occurs, but only with a certain probability (e.g., Baumeister and Lau, 2024).

A common explanation for the fact that only probabilistic rather than invariable cause-effect relationships are observed in experimental psychological studies is the high heterogeneity of psychical phenomena (Hitchcock, 2018). There are various psychological mechanisms in the human psyche, each of which trying to influence behavior in response to an event in its own way and according to its own standards. Since it is always the person’s entire psyche with all its different mechanisms that reacts to an event, a specific psychological mechanism can be isolated from all other psychological mechanisms only in limited ways. To use an analogy: If a feather or a steal ball are placed in a physical vacuum, their usual way of reacting to physical forces will not change. But if one tried to put humans in a “psychological vacuum” in the sense that their inner psychological forces are eliminated (if that were even possible), then they would probably go mad.

For this reason, unlike in the natural sciences, the effects of cause-effect mechanisms that are not the focus of the theory under investigation cannot be completely excluded in experimental psychological studies. Indeed, this fact is also reflected in the quality standards that define the best possible way to conduct experiments in the field of psychology. For instance, in the already mentioned editorial of the journal *Experimental Psychology*, the editors describe the best possible way to conduct experiments as follows (Eder and Frings, 2018, p. 258):

“The design of experimental research should be guided by the *max-con-min* principle: *maximize* the systematic variance of the experimental variables under scrutiny; *control* systematic error variance (or “bias”) induced by confounding variables; and *minimize* random error variance induced by random variables.”

Interestingly, a third category of effects is introduced in addition to the effect of the investigated cause-effect mechanism that is deliberately manipulated and the effects of the cause-effect mechanisms that are tried to be eliminated: there are obviously further effects (i.e., “random variables”) whose causes are unknown, and which thus unpredictably bias the observed effects of the investigated cause-effect mechanism.

From a methodological perspective, this is often not seen as a major problem. The argument is that as long as the unknown cause-effect mechanisms are independent of each other and vary randomly and unsystematically across situations and persons, a specific mechanism can nevertheless be isolated by collecting many observations and averaging across the observations. By doing so, only the mechanism one is interested in causes systematic effects at the level of the averaged observations while the unknown mechanisms cause unsystematic random effects which level each other out. In fact, this is the research logic that almost all experimental studies follow today: a theoretically postulated cause-and-effect mechanism is examined at the level of averaged observations in a sample of individual persons that is supposed to be representative of the population about which the theory makes statements.

However, such a research logic has an often-overlooked consequence regarding the type of phenomena about which knowledge is generated. One often encounters the belief that this type of research would provide knowledge about the occurrence of psychical phenomena at the level of individual persons. However, this belief is actually misleading because the level of observation is not individual persons but averaged observations across individuals. Drawing conclusions from cause-effect relationships observed at the level of averaged observations across individual persons about the existence of cause-effect relationships at the level of individual persons would only make sense if a premise were fulfilled: the individual persons must be homogeneous in terms of the psychological structures and processes producing the observed phenomenon. In this case, how people react on average when they encounter an event would be informative for how an individual person reacts to the event, because the same pattern as observed on average at the group level would show up when an individual person repeatedly encounters the event.

However, numerous research findings call this premise into question, suggesting that heterogeneity instead of homogeneity is a defining characteristic of the functioning of the human psyche (e.g., Richters, 2021). Indeed, what distinguishes the human psyche is precisely that genetically underdetermined psychical structures and processes exist whose functioning parameters are determined by the experiences made in the idiosyncratic physical, social, and cultural environment. Such biographically determined individual adaptation processes can be found right down to the neuronal level. For instance, the experience-dependent elimination of neurons and synapses (“pruning”) is regarded as one of the most important developmental mechanisms that enables the brain to adapt to the demands of the individual environment (e.g., Sakai, 2020).

As can be mathematically shown (i.e., the ergodic theorems), strict conditions would actually have to be met in order to transfer cause-effect relationships observed at the level of averaged observations across persons to the level of an individual person. However, these conditions are almost never checked and actually rarely met in psychological research (Molenaar and Campbell, 2009). This fact is particularly evident in experimental studies in which the behavior of people in real life is studied. For example, it is a common method in the field of educational science to investigate the effect of a learning method in an experiment in which the average performance in a group of people using the learning method is compared with the average performance in another group of people not using the learning method. However, individual performance varies around the averaged performance of the group, which means that the learning method gives some people a stronger advantage, while others have no advantage or possibly even a disadvantage. And since the average performance of the group does not provide any information about whether an individual person’s performance is above or below the average performance, the observation that persons on average benefit from a certain learning method does not allow conclusions to be drawn as to whether the learning method is also effective for a particular individual person.

Given this fact, it is worth pointing out that classic definitions of the field of psychological research actually contain a misleading inaccuracy. For instance, according to the definition of the American Psychological Association, “psychology is the study of the mind and behavior”.^5^ Such definitions give the impression that psychological science studies mind and behavior at the level of individuals. However, since most experimental psychological studies actually explore psychical phenomena at the level of averaged observations across individuals, a more adequate definition would actually have to include the addition “psychological science is the study of the mind and behavior *at the level of averaged observations across individuals*.”

## 6 A possible second limit for the establishment of general theories: the occurrence of an irresolvable uncertainty in psychological research findings

The previous explanations show that there is a fundamental limit to the attempt to establish general theories of the functioning of the human psyche, namely that only probabilistic cause-effect relationships at the level of averaged observations across individuals can be empirically demonstrated. However, several recent studies suggest that the limitations are even more fundamental. The probabilistic cause-effect relationships observed in a specific study should be replicated when the same study is carried out again. However, as shown in several recent studies, this is not the case.

A first indication of a general replication problem emerged in a large-scale attempt to replicate 100 experimental and correlational studies published in high-ranking scientific psychological journals (Open Science Collaboration, 2015). While 97 % of the original studies had reported significant results, only 36 % of the replications had significant results. A similar picture emerged in a recent study where a text-based machine learning model was used to estimate the replication likelihood for more than 14,000 articles in six subfields of psychology published from 2000 to 2019 (Youyou et al., 2023a). The machine learning model was trained on the main texts of 388 manual replication studies in psychology that reported pass/fail replication outcomes to predict a paper’s replicability based on the text in the manuscript. The results suggest that the mean likelihood of successful replication for a published psychological paper is only 0.42 (for criticisms, see Crockett et al., 2023; Mottelson and Kontogiorgos, 2023; for a reply to the criticisms, see Youyou et al., 2023b).

An initial reaction to the replication crisis from the psychological research community was the assumption that questionable research practices (e.g., Simmons et al., 2011), problematic incentive structures (e.g., Fanelli, 2010), and statistical misconceptions (e.g., Greenland et al., 2016) were responsible for the low replication rate, which led to various initiatives to improve the quality of research methods in psychology in order to increase the replication rate (e.g., Korbmacher et al., 2023). However, as shown in the above-mentioned study where the replication likelihood for psychological articles from 2000 to 2019 was estimated (Youyou et al., 2023a), the improvements in method-related and incentive-related problems had only a comparatively small impact. The average replication likelihood had decreased by approximately 10 % from 2000 and 2010 before the replication problem was brought to attention and before the various initiatives were launched. After that, the replication rate returned to the 2000 level and was still below 0.50 in 2019, suggesting that the reason for the problem of empirically demonstrating general regularities in psychology may be more fundamental than only the existence of questionable research practices and problematic incentive structures.

That this is indeed the case is shown by several recent studies which demonstrate that even exactly the same data set does not allow simple-sounding psychological questions to be empirically answered clearly and unambiguously. For instance, in a study by Silberzahn et al. (2018), 29 research teams were asked to empirically answer the question of whether soccer referees are more likely to give red cards to dark-skin-toned players than to light-skin-toned players, based on exactly the same data set. The result pattern showed that the different analysis methods used by the different research teams did not converge. The estimated effect sizes ranged from 0.89 (less likely) to 2.93 (more likely) in odds-ratio units, and neither the teams’ prior beliefs about the effect of interest nor their level of expertise nor the quality of the used methods readily explained the variation in the outcomes of the analyses. A similar finding was reported in a recent study by Breznau et al. (2022), where 73 independent research teams used exactly the same data set to empirically answer the question of whether more immigration will reduce public support for government provision of social policies. Instead of convergence, the results reported by the different research teams varied greatly, ranging from large negative to large positive effects of immigration on public support, and the variance in the obtained results was again not explained by the quality of research methods or the level of expertise.

These findings suggest that even when the problems of questionable research practices and biasing incentive structures are completely removed, and even when exactly the same data set is used when trying to answer a simple-sounding psychological question, it is impossible to establish reliable general theories. Instead, it seems, that there is an uncertainty in psychical phenomena that hampers attempts to establish general theories about the functioning of the human psyche.

## 7 Is basic experimental psychological research also affected by the occurrence of an irresolvable uncertainty?

In the two mentioned studies on the occurrence of an irresolvable uncertainty, psychical phenomena occurring in real-life were examined, which means that numerous mechanisms of the human psyche interact in a variety of ways without any experimental control. It could therefore be hoped that an irresolvable uncertainty will not occur in experiments in the field of basic experimental psychology, where simple psychical phenomena are investigated in artificial laboratory environments under carefully controlled conditions. And indeed, as already described above, this belief is very widespread in this field of research.

However, the results of the studies on the replicability of psychological studies suggest that the problem of uncertainty does also affect experimental studies, thus casting initial doubt on the assumption that the uncertainty observed in psychological studies may disappear in basic experimental psychology. If the use of the experimental method is associated with a lower uncertainty in the observed findings, the replication likelihood should be higher for experimental compared to non-experimental psychological studies. However, in the above-mentioned study (Youyou et al., 2023a) where the replication likelihood for more than 14,000 published psychological studies was estimated, the opposite was observed: the replication likelihood was lower for experimental studies than for non-experimental studies, a finding that was observed for all six subfields of psychology.

This finding suggests that there is a peculiarity in the functioning of the human psyche which entails that even when apparently simple psychical phenomena are explored in artificial laboratory environments under carefully controlled conditions, no law-like behavior can be observed. As already briefly mentioned, a characteristic of the human psyche is that it is a system which consists of numerous components that mutually influence each other and collectively shape the observed psychical phenomena. This type of organization has fundamental consequences for the occurrence of regularity in behavior.

As shown in the domain of chaos research, even if all components of such a system function in a strictly deterministic manner, it is impossible to predict what behavior the system will exhibit when it encounters certain conditions. The reason is that the smallest differences in the initial conditions can build up and alter the behavior of the system, which makes the behavior of the system unpredictable, a phenomenon called deterministic chaotic behavior (for a comprehensive description, see Prigogine and Stengers, 1997). An illustrative example is a pendulum that swings back and forth over two magnets. Unless the pendulum is not released directly over one of the two magnets, it is impossible to predict over which magnet the pendulum will come to rest, because minimal and no longer measurable shifts in the starting position of the pendulum can lead to different end positions. The phenomenon of chaotic behavior has entered everyday language in figurative form of the so-called “butterfly effect,” which refers to the hypothetical assumption that large-scale phenomena such as tornados can be influenced by such small differences in the initial conditions as the flapping of a butterfly’s wings (for a discussion, see Pielke et al., 2024).

The occurrence of chaotic behavior in systems consisting of mutually influencing components suggests that the assumption that law-like behavior can be observed when simple psychical phenomena are explored in highly controlled experimental settings may not be true. Given that in such systems as the human psyche, minimal differences in the initial conditions can lead to large and unpredictable differences in the observed behavior, it could be that even when exploring apparent simple psychical phenomena in an experimental setting with careful control of unwanted cause-effect mechanisms, still an irresolvable uncertainty occurs because the observed phenomena unpredictably vary as a function of minimal, and from the perspective of the investigated research question irrelevant, details of the experimental setting.

A closer look at the inner organization of the human psyche reveals another possible reason why even the apparently simple psychical phenomena that are explored in the field of basic experimental psychology may not show regularities that can be described by general laws. What distinguishes the human psyche from mechanistic systems such as a pendulum swinging over two magnets is that the inner components not only mutually influence each other. In addition, the inner components are additionally organized within a multi-layered structure of ascending levels of increasing organizational complexity. The special characteristic of such complex systems^6^ is that at the higher levels of organization, novel phenomena with novel properties emerge that do not exist at the lower levels. The emergent phenomena on the higher levels in turn influence the functioning of the mechanisms on the lower levels in order to make them subserve the objectives pursued at the higher levels (e.g., Feinberg and Mallatt, 2020).

An illustrative example is the phenomenon of the experience of emotions. One of the most common definitions defines emotions as episodes of interrelated, synchronized changes in the states of all five organismic subsystems (cognitive, neurophysiological, motivational, motor expression, subjective feeling) in response to the evaluation of an external or internal stimulus event as relevant to major objectives of the organism (Scherer, 2005). Emotions therefore emerge on a higher organizational level in the sense of an organizational structure which provides various cross-system reaction patterns, and the mechanisms on the lower levels change depending on which emotion is currently experienced on the higher level.

The example of the higher-level mechanism of experiencing emotions illustrates why it makes no sense to postulate that the functioning of a low-level mechanism can be described by a general law if the mechanism is an integrative part of a complexly organized system. Since in such systems the concrete operating principles of the lower-level mechanisms are determined by the phenomena occurring on the higher levels, there is simply no general operating principle that could be described by a general law. For instance, it makes no sense to claim that the functioning of iconic memory, which is considered one of the basic cognitive processes of the human psyche, can be described by a general law because studies show that the properties of iconic memory vary as a function of the emotions currently experienced at the higher level of organization (e.g., Kuhbandner et al., 2011a,b). And since people are in a certain emotional state at every time point in their lives, it makes no sense to claim that the properties of iconic memory can be explained by a general law.

One could still hope that it may at least be possible to observe general regularities for certain interactions between low-level and high-level mechanisms. For instance, it could be that although the properties of iconic memory cannot be described in the form of a general law, the respective functioning in a certain emotional state can be described in the form of a general law. However, this hope is dashed by another peculiarity of the human psyche. What characterizes the human psyche is not only that its components are organized within a multi-layered structure of ascending levels of increasing organizational complexity. As already briefly mentioned above, what makes the human psyche special is that the psychical structures and processes at the higher levels are not genetically fixed but idiosyncratically developed based on the physical, social, and cultural environment of a particular individual. The consequence is that there is no general regularity that can be described by means of general theories because the functioning of lower-level mechanisms changes as a function of higher-level mechanisms which do not function in mechanistic ways but in idiosyncratic ways.

An illustrative example is the attempt to establish the functioning of emotions empirically. Initially, emotion research was guided by the hypothesis that each emotion has its own essence, that is that each emotion can be described by a separate mechanism that is specific to that emotion. If this were the case, each emotion would follow a certain general law, which could then be empirically proven. However, after hundreds of studies, the picture that has emerged is completely different. Both at the level of the facial, the cognitive, the motivational, the physiological, and the neuronal level, tremendous variation both within and across emotional categories is observed across studies, even when the same methods, stimuli, and sampling from the same population of participants were used, a pattern of finding that has been summarized in an overview in the statement “variation is the norm is a fair summary of the experimental literature on emotion” (Barrett and Westlin, 2021). In view of this variability, a new theoretical framework has been established that assumes that emotions have no essence but are categories of variable instances that vary from context to context depending on what has been functional according to the past experiences of a person (Barrett, 2013).

## 8 Is there a “butterfly effect” in basic experimental psychological experiments?

The described characteristics of the functioning of the human psyche suggest that a previously overlooked problem could exist in the field of basic experimental psychology. It could be that even when examining apparently very simple psychical phenomena under apparently highly controlled laboratory conditions, no regularity in behavior can be observed. Although attempts are made to tailor the experimental situation as closely as possible to the cause-effect mechanism under investigation, there are numerous details of the experimental situation which are often unintentionally chosen because they appear to be irrelevant for the mechanism under investigation (e.g., the concrete color of stimuli, the concrete spatial and sequential arrangement of stimuli, the current affective state of a specific subject). However, because in complex systems such as the human psyche, even minimal, and from the perspective of the investigated research question irrelevant, details of the experimental situation or the internal state of the participants can have large and unpredictable effects, the effect observed in a particular experiment may actually not reflect a generalizable effect of the cause-effect mechanism that is purportedly investigated, but actually the effects of minor details that unintentionally occurred in this specific experiment. In particular, even if one tries to explicitly control the effect of such minor details, this may not necessarily solve this problem if the mechanism of interest actually systematically varies as a function of these details.

A look at various research paradigms in the field of basic experimental psychology shows that it indeed often turns out that initially obtained findings actually depend on minor details that were unintentionally chosen in the initial experiments. For instance, in research on visual memory, the colors of visual objects are typically unintentionally chosen by experimenters. However, as shown in a series of studies, basic processes such as color-form binding are not uniform processes that work the same for all types of colors. Instead, red colors are particularly strongly bound whereas green colors are particularly weakly bound (Kuhbandner et al., 2015a).

Such effects of the occurrence of uncertainty due to the use of different types of stimuli have led to some of the initially postulated laws of the human psyche proving to be untenable. For example, Miller’s law on the capacity of working memory, mentioned at the beginning, was in view of numerous contradictory findings described as “the legend of the magical number seven” (Cowan et al., 2007), and replaced by the “magical number four,” which seemed to better describe the regularities observed across experiments (Cowan, 2010). However, meanwhile the variation in the observed findings is so huge that neither the magic number seven nor the magic number four can satisfactorily describe the psychical phenomena occurring in studies on working memory. Instead, it was suggested to abandon the theory of a fixed capacity in favor of theories that postulate that the quantity of items that can be held in working memory depends on the precision of the stored representations, with humans being able to flexibly trade between quantity and precision depending on context such as the currently experienced emotions (e.g., Spachtholz et al., 2014).

The case that further research reveals that initially obtained effects turn out to be effects that are actually tied to minor details of the experimental situation is found not only at the level of the stimuli chosen in an initial study, but also at the level of the response format chosen. For instance, in a highly cited study on the capacity of visual long-term memory, a remarkably high ability was observed to discriminate previously seen objects from highly similar new objects, which led the authors to conclude that visual long-term memory has a massive storage capacity for object details (Brady et al., 2008). However, in subsequent research, it turned out that this ability strongly varies as a function of subtle details of the test used. Performance is remarkably high when a test is used where the object previously seen and the new object are presented simultaneously on the screen (two-alternative forced-choice recognition test), but not when a test is used where the two objects are shown individually on separate screens (old-new recognition test; Cunningham et al., 2015).

Complicating matters even further, it turned out that even when consistently using two-alternative forced-choice recognition tests, a convergent result pattern is not necessarily observed. An example is the research on the phenomenon of recognition without awareness. An initial study showed that when testing recognition for highly complex visual stimuli with a two-alternative forced choice recognition test, recognition performance was highly accurate although the subjects reported that they had the feeling of being unable to remember the stimuli (Voss et al., 2008). However, another research team was not able to replicate this finding although the original study was reproduced as closely as possible (i.e., the same stimulus set, the same stimulus presentation times, etc.), concluding that recognition without awareness is an elusive phenomenon (Jeneson et al., 2010). As it turned out, the reason of this inconsistency across experiments was a slight variation in the way the subjects were instructed, encouraging subjects to guess in one case and to respond more confident in the other case (Voss and Paller, 2010).

In addition to effects of minor details of the experimental setting used in a particular study, further effects arise from minor details of the environment in which a particular experiment is carried out. For instance, it has been shown that the results obtained with exactly the same experimental setting vary as a function of environmental factors such as the sex or the attire of the experimenter (e.g., Green et al., 2005) or the body posture of the subjects (e.g., Muehlhan et al., 2014), the latter being one of the main factors why findings obtained in non-imaging standard laboratory settings, where subjects typically perform experimental tasks sitting upright, are sometimes difficult to replicate in neuroimaging settings, where subjects typically perform experimental tasks lying in supine position.

However, even when exactly the same experimental task is performed by subjects in exactly the same laboratory setting, the obtained results can unsystematically vary across the participating subjects. For instance, a prominent theory in the phenomenon area of attention is based on the assumption that attention can be allocated advantageously to specific objects in visual space, an ability called object-based attention (e.g., Watson and Kramer, 1999). However, it turned out that such effects were difficult to replicate. In a comprehensive attempt to resolve the confusion reported in previous studies (Pilz et al., 2012), it was on the one hand shown that the occurrence of such effects depends on minor details: object-based attention effects were only observed when the stimuli were arranged horizontally but not when they were arranged vertically. However, even worse, bootstrapping showed that object-based attention effects were not observed in all of the tested subjects but only in a small minority of the subjects. The authors conclude that computing averages across tested subjects in experiments may not be a suitable method to create theories of cognition and perception because the variation on the level of individual subjects has to be taken into account for a true understanding of how cognition and perception work.

Critically, the effects of minor details of the experimental situation that are initially erroneously viewed as irrelevant can be so subtle that a whole research community does not notice this, creating the wrong impression that there is a general theory although this is actually not the case. Such a case can occur when all of the studies conducted to test a general theory consistently use the same specific research method which actually represents a special case, without the research community noticing this fact. An example is the so-called motivational-compatibility effect, which assumes that for positive stimuli approach behavior is faster elicited than withdrawal behavior, whereas for negative stimuli withdrawal behavior is faster elicited than approach behavior. In countless studies in which subjects were presented random series of positive and negative stimuli and their response speed and frequency of errors for approach and avoidance behavior measured, such an effect seemed to occur consistently over and over again (for a meta-analysis, see Phaf et al., 2014).

However, it turned out that a hidden confounding variable at the level of a minor detail of the experimental situation was present in all of the studies of this type. As shown in a series of studies, in such experiments, strong valence-independent trial-by-trial effects are observed because switching from approach to withdrawal behavior is much easier than vice versa (Kuhbandner et al., 2015b). These asymmetrical switch costs strongly biased the observed effects on trials where the opposite behavior had to be shown in the previous trial, creating the illusion that there is a similar motivational-compatibility effect for both negative and positive stimuli. However, looking only at the trials that were not biased by these asymmetrical switch costs revealed that motivational-compatibility effects are actually largely absent for negative stimuli and much stronger for positive stimuli. It is also interesting that this study, despite being published in a topic-relevant journal (*Cognition and Emotion*), has not been cited once yet by the motivational-compatibility effect research community, and that, to our knowledge, no study has taken this fact into account to date, which demonstrates how immune research communities can be to methodological problems.

There is also the particularly problematic case where details of the experimental situation that later turn out to be relevant are initially considered so irrelevant that they are even not described in the methods section of studies. This case is particularly problematic because by reading just the methods section of a study one cannot conclude that these boundary conditions even may exist. A prominent example are the studies on the electrophysiological correlates of attention and memory by the famous EEG researcher Steven Luck (e.g., Luck, 2012). In his textbook on the event-related potential technique (Luck, 2014), there is a box at the end entitled “Keeping subjects happy,” which describes how Luck treats his subjects in the laboratory: he tries to keep them happy by playing their favorite music throughout the whole experiment, noting that the music brought by his subjects included all kinds of genres from classical, pop, rock, metal, rap, country, electronic, ambient, and just about everything else imaginable. However, in his published scientific papers, this treatment of subjects is not mentioned. Obviously, he assumes that the affective state of a subject is irrelevant for the basic cognitive processes he is investigating.

However, as it turned out, basic cognitive processes and their electrophysiological correlates vary not only quantitatively but even qualitatively as a function of the affective state of a subject. For instance, when making participants happy by playing happy music and asking them to retrieve happy memories, visual objects are stored in the form of coherent object representations mediated by attention-related brain activities. By contrast, when making participants sad by playing sad music and asking them to retrieve sad memories, visual objects are stored in the form of independent feature representations mediated by preattentive brain activities (Spachtholz and Kuhbandner, 2017).

Finally, there is also the case where a theoretically postulated psychical mechanism is confirmed in numerous experiments, but it turns out that the regularity observed in the experiments has nothing to do with the postulated psychological mechanism itself but is actually the effect of a minor detail of the experimental situation, which was unintentionally kept the same in all experiments. An example is the research on the so-called anger-superiority hypothesis, according to which it is easier to detect angry faces than happy faces in a crowd of neutral ones. The possible existence of such an effect was initially suggested using pictures of real faces (Hansen and Hansen, 1988). In response to criticism that the observed effect might not be due to emotional causes but due to differences in low-level visual features, subsequent studies used line drawings of emotional faces that consisted of identical features that were just spatially aligned differently (e.g., using the same curved line for the mouth, only oriented upwards versus downwards; Oehman et al., 2001). However, there was still criticism that the presentation of upward or downward curved lines alone could be sufficient for the effect to occur, which was in fact shown in follow-up studies (Coelho et al., 2010).

This finding indicates that the postulated psychological mechanism of an alleged superiority of angry faces, which was initially viewed as empirically proven, was actually driven by an emotion-independent minimal detail of the experimental situation. More generally viewed, as shown in more recent meta-analyses, the research history of the anger-superiority hypothesis is another example where, as research into the phenomenon increases, it turns out that the initial hypothesis of a general pattern breaks down into many individual findings that can no longer be summarized in the form of a general theory. For example, the authors of a recently published meta-analysis on the electrophysiological correlates of the anger superiority effect conclude in the abstract (Liu et al., 2021, p. 1): “the mean effect size difference between angry and happy expressions was ns. N2pc effect sizes were moderated by sample age, number of trials, and nature of facial images used (schematic vs. real) […]. As such, possible adaptive advantages of biases in orienting toward both anger and happy expressions warrant consideration in revisions of related theory.”

## 9 Possible solutions and resulting consequences

As shown in the previous section, the assumption prevalent in the field of basic experimental psychology that law-like behavior can be observed if the complexity of the human psyche is reduced by creating experimental settings in which apparently simple psychical phenomena occur under apparently highly controlled conditions is often not fulfilled. The reason is that a special property of complex systems such as the human psyche is ignored in current research practice, namely that minor and, from the perspective of the investigated research question, irrelevant details of the experimental situation or the internal state of the participants can produce large effects. This leads to the accumulation of many individual experimental findings which, however, do not contribute to a cumulative acquisition of knowledge due to the occurrence of an unsystematic variability across the individual findings.

The question of possible solutions to this problem seems to be at first glance easy to answer: law-like behavior in experiments can only occur if a postulated cause-effect mechanism is studied in a truly isolated way. In this case, even the smallest differences that are irrelevant from the perspective of the investigated research question can no longer produce any effects. However, this necessary precondition for the possibility of the occurrence of law-like behavior is accompanied by fundamental consequences for the intention to explore the human psyche with the experimental method.

### 9.1 Consequences at the level of theory

The precondition that a cause-effect mechanism must be studied in a truly isolated way is accompanied by certain requirements at the level of the theoretical concepts based on which cause-effect relationships are formulated. As a starting point for working out these requirements, it is first necessary to clarify what exactly is meant by the term “concept”. Building on this, it is then necessary to work out what special features theoretical concepts should have so that an experimentally isolatable cause-effect mechanism can be postulated based on them.

From a philosophy of science perspective, one fundamental assumption is that concepts are products of the human psyche, which allow humans to abstract from the abundance of internally representable entities. This abstraction is achieved by assigning entities that can actually be distinguished from each other to an overarching common concept, which defines a property that characterizes the set of entities assigned to the concept. An illustrative example is the concept “red,” which is an overarching property that represents as a common concept all of the actually different hues that belong to this concept. Another example is the concept “intelligence,” which is an overarching property that represents as a common concept the entirety of a person’s problem-solving abilities. As the examples of the concepts “red” and “intelligence” illustrate, concepts can never be directly observed as such. Instead of seeing “red” or “intelligence,” we can only ever see the individual referents (i.e., the currently perceived hue or the currently observed problem-solving ability) that we have agreed on belong to the concepts of “red” or “intelligence.”

With regard to the question of what special features theoretical concepts should have so that an experimentally isolatable cause-effect mechanism can be formulated based on them, a straightforward requirement is that the referents of a concept must be precisely defined. If this is not the case, degrees of freedom arise with respect to the determination of the details of the experimental setting, which creates room for the occurrence of an irresolvable uncertainty. This requirement can be well illustrated by comparing the characteristics of everyday language terms and scientific terms, as done in the following quote from Bischof (2014, p. 37; translated by the authors):.

When we talk about psychical matters in everyday life, we use everyday language. These terms are strange creatures: blurred fields of meaning, knotted associations of fragments of ideas that condense around a core and run out towards the edge without clear boundaries. It is easy to say what a ‘mountain’ is near the summit. But where does it end, where does the ‘valley’ begin? What is the minimum number of hairs a ‘brush’ must have? (...) Scientists sometimes make use of the words found in everyday language. They speak, for example, of ‘power’ or ‘work’ or ‘performance’. But they subject the concepts that such words are supposed to denote to a rigorous definition. They nail down their exact referents and excludes everything else.

Problematically, the theoretical concepts used in psychological theories often do not do justice to the requirement that the referents of a concept must be precisely defined (for a recent discussion of this problem, see Hutmacher and Franz, 2024). Instead, to quote Norbert Bischof again,

one often avoids clear definitions, relying on one’s everyday feeling for language; the terms are left unpurified in their cloud of unclear connotations, and so that this is not noticed so quickly, at least the everyday expression is replaced by a technical term (Bischof, 2014, p. 38; translated by the authors).

By doing so, only the illusion is created that the concepts on which a psychological theory is based are precisely defined, although in reality a hidden universe of uncertainty is introduced.

However, the use of precisely defined theoretical concepts is not sufficient to enable a true isolation of cause-effect mechanisms in experiments. This can be illustrated using the example of the concept “intelligence.” If one defines “intelligence” as the entirety of a person’s problem-solving abilities, and if it were the case that all existing problem-solving abilities are known, then the concept would be absolutely precisely defined. However, if one were to formulate a cause-effect mechanism based on a concept such as “intelligence” and attempt to isolate this mechanism in an experiment, this would be an impossible undertaking.

The reason for this has to do with a special property of concepts. Concepts can abstract from the abundance of internally representable entities with a low or high degree of abstraction. At the lowest level of abstraction, the referents of a concept are entities that each are concretely perceivable at a given moment. An example is the concept “red” which refers to the group of perceivable colors with a specific hue. Such low-level concepts are characterized by an unidimensional structure because each of the referents carries the property defined at the concept level completely within itself (e.g., unidimensional concepts).

At the higher levels of abstraction, the referents of a concept are not entities that each are concretely perceivable at a given moment, but other concepts that are located at a lower level of abstraction. This ability enables humans to flexibly represent the complexities of the world and the psyche at increasingly higher levels of abstraction with increasingly broader concepts. An example is the concept “intelligence.” For instance, at a lower level of abstraction, verbal working memory abilities and visual working memory abilities can be distinguished because they are each based on independent mechanisms. These abilities can be represented at the next higher level as a joint entity by assigning them to the broader concept “working memory ability.” At the next higher level, the referents of the concept “working memory ability” can be assigned to the broader concept of “fluid intelligence,” which represents as a joint entity all abilities that share the common feature that they are independent of previously acquired knowledge. And finally, the referents of the concept “fluid intelligence” can be assigned to the broader concept of “intelligence,” which represents as a joint entity the entirety of a person’s abilities, including the abilities that depend on previously acquires knowledge. Such higher-level concepts are characterized by a multidimensional structure because each of the referents represents only a part of the property that is defined at the level of the higher-level concept.

With regard to the attempt to truly isolate cause-effects mechanisms in experiments, theoretical descriptions based on higher-level multidimensional concepts such as intelligence are problematic. Multidimensional concepts do not represent a concrete mechanism that may exist in reality. Instead, they are aggregates of different mechanisms that are actually each represented by their own concepts at a lower level of abstraction. For instance, the concept “working memory” does actually not represent a concrete mechanism. Instead, this concept summarizes the results of the separate systems of verbal working memory and visual working memory, which each function based on their own principles. Consequently, although multidimensional concepts such as “intelligence” can be precisely defined, they do not allow to exactly specify which mechanism should be isolated in a concrete experiment because different mechanisms are represented as a joint entity, which leads to the occurrence of an unresolvable uncertainty. Accordingly, a necessary precondition for the occurrence of law-like behavior in experiments is not only that the examined theoretical concepts are precisely defined but also that they are unidimensional low-level concepts.

Problematically, however, the use of broad multidimensional concepts is common in current basic experimental psychology. This creates the illusion that the same cause-effect mechanism is examined in different experiments, although actually different operationalizations of the same multidimensional concept were implemented. An illustrative example is the experimental research on “attention” and “working memory.” There are hundreds of studies that are either framed under the theoretical term “attention” or the theoretical term “working memory,” which gives the impression that there exist two independent low-level psychological mechanisms within the human psyche, each with its own independent mode of functioning. However, if one were to look at the definitions found in typical studies on “attention” and “working memory,” one might come to the conclusion that these two terms have actually a strongly overlapping range of meaning. For instance, “working memory” is commonly defined as the mechanisms that hold the information currently most relevant for an ongoing behavior available for processing (e.g., Oberauer, 2019), and “attention” is commonly defined as the mechanisms that select, modulate, and sustain focus on information currently most relevant for an ongoing behavior (e.g., Chun et al., 2011). And if one were then to set out to explore the respective meanings more deeply, a whole universe of interconnected lower-level mechanisms would open up (for such an attempt, see, e.g., Oberauer, 2019), all of which would actually have to be described separately in a theoretically more fine-grained way if experimental psychological research is to be conducted in a meaningful way.

### 9.2 Principal limitations

As shown, it is a necessary precondition for the occurrence of law-like behavior in experiments that the explanatory concepts used in the examined theory are precisely defined unidimensional low-level concepts. This fact results in a fundamental limitation as to which types of psychical phenomena can be meaningfully investigated using the experimental method.

As already briefly mentioned, precisely the ability to build broad and abstract mental concepts that allow to represent the complexity of the world in a non-complex way is one of the central functional principles of the human psyche. In fact, it is exactly this ability that allows humans to show stable behavior in a situation where normally no stability occurs due to the occurrence of deterministic chaos. To establish order in this chaos, higher-level psychological mechanisms had to be established which operate on concepts that abstract from the vast number of details that are actually distinguishable on the lower levels of abstraction, but whose distinguishability is unimportant from the perspective of the acting person (for a detailed model, see, e.g., Tononi, 2012).

Accordingly, there is a first fundamental limitation: From the fact that law-like behavior in experiments can only occur if an investigated cause-effect relationship is based on precisely defined explanatory concepts with a low degree of abstraction, and from the fact that it is precisely the characteristic of higher-level mechanisms of the human psyche that they function based on fuzzily defined concepts with a high degree of abstraction, it follows that the higher-level mechanisms of the human psyche cannot be meaningfully investigated using the experimental method.

However, there is a second fundamental limitation preventing the occurrence of law-like behavior in experiments even when precisely defined low-level mechanisms are examined: the functioning of a low-level mechanism must not vary as a function of states at the higher level of the human psyche. As described above, if this is the case, it makes no sense to postulate that the functioning of a mechanism follows a general rule because there simply is no general rule. The ignoring of this fact often leads to the occurrence of unfruitful discussions in experimental psychology. An illustrative example is the history of research on the question of how the features of visual objects are stored in memory. In two simultaneously published papers, contrasting findings were observed. The findings of a study by Utochkin and Brady (2020) suggested that objects are stored as unbound feature representations. By contrast, the findings of a study by Balaban et al. (2019) suggested that objects are stored as feature-bound object representations. A common reaction to such contradictory findings is to conclude that more research is needed to clarify which of the two possibilities is correct. However, a more fruitful research strategy that was not considered in either of the two studies is to investigate whether the way the features of visual objects are stored in memory depends on higher-level psychological mechanisms. And in fact, it was shown that the way the features of objects are stored in memory does not follow a general law but qualitatively varies as a function of the emotional state of observers (Spachtholz and Kuhbandner, 2017).

### 9.3 Practical limitations

In summary, it can therefore be said that only a very specific type of psychological mechanisms can be meaningfully investigated using the experimental method, namely low-level mechanisms that function independently of the higher-level mechanisms. It is disputed whether such mechanisms even exist in the human psyche. On the one hand, hundreds of studies claim to have shown that higher-level states such as beliefs, desires, emotions, motivations, intentions, and linguistic representations exert top-down influences on low-level perceptual mechanisms, suggesting that low-level mechanisms that function independently of the higher-level mechanisms do not exist. However, it has been argued that actually none of these studies provides compelling evidence for true top-down effects on perception (Firestone and Scholl, 2016), suggesting that such low-level mechanisms may exist.

However, even if low-level mechanisms exist in the human psyche that function independently of the higher-level mechanisms, there is an additional practical limitation: it is extremely difficult to create experimental situations in which psychical phenomena occur that exclusively reflect the effect of such a low-level mechanism. The reason is that the higher-level mechanisms of the psyche nevertheless influence behavior, even if the mechanism under investigation functions independently of these mechanisms. For example, subjects typically think about what is actually being investigated, how their performance compares to others, how they could improve their performance, or just what is for lunch, which brings additional effects into play that do not necessarily influence the functioning of the mechanism under investigation, but nevertheless influence the behavior observed in an experiment.

A recent study on the capacity of visual working memory shows that such effects even occur in very simple experimental settings (Laybourn et al., 2022). In that study, participants were asked to verbalize any feelings or thoughts they are experiencing while performing a standard visual working memory task where participants were asked to remember simple colored squares. The results showed that a variety of thoughts occurred that substantially varied across participants. For example, some participants perceived the task as meaningless, others perceived the task as a game, while still others perceived the task as an exam situation. Out of the 19 participants, six participants reported a change in motivation, stating for instance that the performance achieved became less and less important for them over time and that they just clicked somewhere on the screen, and three participants stated that they tried different strategies to improve performance. These findings show that even in very simple experimental situations, it cannot simply be assumed that exactly the same psychological mechanism is active in all participants. The authors themselves sum up this problem very well:

“As researchers, we would like participants to be more like machines sometimes, so we can examine their “hardware” most accurately. However, it seems that human functioning is more complex” (p. 1602).

## 10 Consequences for the aim of gaining useful knowledge to explain human behavior by the experimental method

In summary, the present paper shows that there is a fundamental limit to understanding the functioning of the human psyche by means of the experimental method: law-like behavior can only occur in experiments when precisely defined low-level mechanisms are investigated that function completely independent of the higher-level mechanisms of the human psyche. This raises a fundamental question: to what extent can the experimental method be used to gain knowledge that is useful for explaining human behavior?

In order to answer this question, the term “behavior” needs to be broken down in more detail. A first necessary distinction concerns the distinction between the explanation of behavior shown *in laboratory settings* and behavior shown *in real life* (i.e., the so-called ‘real-world or the lab’-dilemma, for a discussion, see Holleman et al., 2020). If human behavior in a laboratory setting is to be explained in which a psychical phenomenon occurs that reflects the effect of a truly low-level mechanism that is truly isolated from all other mechanisms of the human psyche, knowledge gained from experimental psychological research can be helpful. However, if the human behavior in real life is to be explained, knowledge gained from experimental psychology has no explanatory power because the behavior shown in real life is never solely determined by the isolated effect of a low-level mechanism. Instead, in real life the human psyche with all its mechanisms always reacts to situations as a whole, with situations being sometimes even actively created by the human psyche in the first place.

However, the psychological knowledge that can be gained by means of the experimental method is not completely irrelevant for the aim to explain the behavior of humans in real life as sometimes claimed (e.g., Debrouwere and Rosseel, 2022). In order to see this point, a further distinction is necessary with regard to the term “behavior”: the distinction between the explanation of *mechanistic* behavior and *motivated* behavior. This distinction can be illustrated using the following instruction:

“Dear reader, please raise your hand!”

Let us assume that you as a reader have actually raised your hand. If one wants to explain this behavior, one can first take a neuroscientific perspective. And from this perspective, one will come to the conclusion that the raising of the hand was caused by an activation of the area in the brain that controls the hand movement. And from this perspective, one might even find oneself thinking that this brain activation fully explains the behavior, because whenever this brain activation is observed in a person, they always raise their hand. However, although this is a truly causal explanation, it has no explanatory power whatsoever with regard to the question of why someone raised their hand. The actual cause why you as a reader raised your hand was the instruction that we as authors gave, and which you understood and followed. And that we wrote this instruction was of course also caused by an activation of our brains. But again, this does not provide an explanation, because the idea to give such an instruction in our paper came at the end of a long chain of thoughts that have built up in us over many years. And whether you as a reader really raised your hand in response to this instruction depends on whether you were motivated to follow this instruction. And that, in turn, depends on the individual views, beliefs and values that have built up over the years on your higher levels of the human psyche.

Accordingly, when one aims to explain an observed behavior, such as raising a hand, there are two separate types of knowledge which are necessary to explain the behavior. On the one hand, knowledge is needed about the mechanisms which underly the *general* ability to mechanistically react to certain sensory experiences with certain motor responses, regardless of when and under what motivational circumstances the behavior is actually shown (i.e., mechanistic behavior). On the other hand, knowledge is needed about the mechanisms which motivate a particular person to actually exhibit in a particular situation the motor behavior of which they are potentially capable (i.e., motivated behavior). And with the experimental method, helpful knowledge can be gained for the explanation of mechanistic behavior, but not for the explanation of motivated behavior.

Accordingly, experimentally gained knowledge can be important to explain behavior in real life in the sense that someone must have the *general* ability to perform a certain behavior in order to be able to show this behavior as a response. However, if one wants to understand when and under what circumstances a person shows a behavior in real life, knowledge gained from experimental psychology is not helpful. In this case, the question is about why a person is motivated to show a certain behavior, a question that can only be answered based on knowledge about the non-mechanistic higher-level processes of the human psyche which give meaning and direction to a person’s behavior in real life – knowledge that cannot be gained by means of the experimental method.

There is a final important point that needs to be made. Someone could come up with the idea that the occurrence of regular behavior in experiments can also be achieved by setting the states of the tested participants on all levels of the human psyche exactly the same, except for the specific mechanism being investigated. However, if this were at all possible (for a critical discussion, see, e.g., Smedslund, 2016), one would be introducing a hidden assumption about the functioning of the human psyche, namely that it is possible to generalize the functioning of higher-level mechanisms across different people.

However, it is exactly the opposite that constitutes the special characteristic of the higher-level mechanisms of the human psyche. There is no general rule as to how the complexity of the world should ideally be mapped into broad and fuzzy mental concepts on the higher levels. Instead, the optimal granularity with which the world is conceptualized varies idiosyncratically as a function of the current external and internal context and the historical, cultural, and biographical background of an individual observer. If one wants to understand the uniqueness of the human psyche, methods have to be used that take into account the idiosyncratic functioning of the human psyche (for an overview, see Salvatore and Valsiner, 2023). Otherwise, if one were to try to make all people the same in an experiment, one would actually take away exactly what makes humans different from inanimate objects: that humans can react to exactly the same physical situation in unique ways.

## Data Availability

The original contributions presented in the study are included in the article/supplementary material, further inquiries can be directed to the corresponding author/s.

## Footnotes

^1^Here, the term “experimental method” always refers to the use of experiments as a scientific method for establishing general laws.

^2^ https://www.britannica.com/science/science

^3^In the following, the term “general theory” always refers to theories that predict certain cause-effect relationships at the level of an object property which should apply to all objects that have this property.

^4^Following a suggestion by Uher (2021), this article uses the term “psychical” when referring to the phenomena that are explored, and the term “psychological” when referring to the means used for the exploration of a phenomenon.

^5^ https://www.apa.org/support/about-apa

^6^The term “complex system” is not used uniformly in the literature (for an overview, see Ladyman et al., 2013). In the present article, we use this term as an umbrella term to describe systems in which emergent phenomena occur from a collection of interacting parts.
